# Are realist randomised controlled trials possible? A reflection on the INCLUSIVE evaluation of a whole-school, bullying-prevention intervention

**DOI:** 10.1186/s13063-021-05976-1

**Published:** 2022-01-28

**Authors:** Emily A. Warren, G. J. Melendez-Torres, Chris Bonell

**Affiliations:** 1 Department of Public Health, Environments and Society, Faculty of Public Health and Policy, London School of Hygiene & Tropical Medicine, 15-17 Tavistock Place, London, WC1H 9SH UK; 2 Institute of Health Research, College of Medicine and Health, University of Exeter, Exeter, UK

**Keywords:** RCTs, Realist evaluation, Randomised trials, Complex interventions, Adolescent health, Bullying, School environment

## Abstract

We previously proposed that realist randomised controlled trials could be used to evaluate how, for whom and under what conditions complex interventions can be used to activate mechanisms to improve health. While this idea was accepted by some, it was also met with resistance, particularly from some realist evaluators who believe that trials are inextricably positivist and dependent on constant conjunctions to understand causation, and that realist trials are unfeasible because participants and contexts will be insufficiently diverse to enable the testing of context-mechanism-outcome configurations. In this paper, we reflect on analyses of qualitative and quantitative data from the Initiating Change Locally in Bullying and Aggression through the School Environment (INCLSUIVE) trial, and whether these are useful and aligned with realism. We summarise the concerns expressed by realists and reflect on the philosophical and practical challenges that we encountered and whether or not they are related to the trial’s design. Finally, we reflect on the trial’s weaknesses and highlight areas that future researchers might consider when running realist trials. We conclude that realist randomised controlled trials are philosophically coherent, practically feasible, and can produce nuanced findings.

## Introduction

In 2012, the INCLUSIVE trial was presented as being the first realist randomised controlled trial (RCT) [1]. Realist trials aim to examine how intervention resources introduced into various contexts enable the activation of contextually contingent mechanisms which generate improvements in health, and assess how those vary by context. Realist evaluation within RCTs should minimise bias and confounding in these analyses of effects. However, proposals for realist trials have been met with concerns about the philosophical compatibility between realism and RCTs, and practical concerns, particularly in relation to the assessment of mechanisms and whether sufficiently diverse settings can be included to test context-mechanism-outcome configurations (CMOCs). Since this debate began, we have completed our trial [2] and continue to analyse data from it. In this paper, we reflect on how we engaged with realist evaluation, what challenges we faced and whether or not we were able to conduct an informative realist RCT.

The INCLUSIVE trial evaluated Learning Together (LT), a complex, whole-school intervention that provided resources intended to enable secondary schools to reduce bullying and aggression. These resources included an intervention manual, an annual needs assessment report (NAR) generated from an annual student survey, a social and emotional learning curriculum, an external facilitator in the first 2 years of the intervention and training on restorative practice (a half-day training introducing restorative practices for all staff members and a 3-day intensive training for selected staff). These resources were provided to enable the following school processes: convening an action group with at least six students and six staff members to meet at least once every half-term; this group reviewing rules and policies to ensure they were supportive of restorative practices; this group deciding and implementing local actions based on the NAR; staff using restorative practice to address student conflict or misbehaviour (convening meetings between bullies and victims to understand the source of problems, give the victim the opportunity to explain how they feel and give the bully the opportunity to listen, and make amends); and staff teaching the curriculum.

The theory of change for LT was informed by a mid-range theory known as the theory of human functioning and school organisation [3]. This proposes that schools have an instructional order (concerning academic learning) and regulatory order (concerning the social norms, behavioural expectations and shared values). Commitment to these orders can be increased by “reframing” school practices on student needs and “eroding” the boundaries that separate students from staff, student groups from each other, students’ intellectual learning from their personal development and the school from the surrounding community. The theory proposes that reframing and boundary erosion will particularly engender the commitment of students from deprived backgrounds for whom school cultures may be particularly alienating and for whom engagement with education may be more challenging. Building commitment to school will, the theory proposes, equip students with the skills and social relationships so that they avoid participation in anti-school groups and risk-taking behaviours [4, 5].

Informed by this mid-range theory, LT’s theory of change proposes that the intervention resources will help schools build student commitment via reframing school practices around student needs and eroding boundaries between staff/students and different areas of the curriculum. This is theorised as being achieved via the action groups bringing staff and students together in a constructive environment to make collaborative decisions and improve school practices, restorative practices focusing on students’ needs for genuine conflict resolution and the curriculum addressing student needs for social and emotional skills and eroding the boundaries between students’ personal development and academic learning.

INCLUSIVE was a 3-year cluster RCT of the LT intervention including a mixed-method process evaluation [1, 6], designed to be the first realist RCT [1]. The trial aimed to embrace realist approaches by using qualitative research to assess, refine and augment the starting theory of change and inform a number of CMOCs and using moderation and mediation analyses to test these CMOCs. These ideas were quickly incorporated into the Medical Research Council’s guidelines on process evaluations for complex interventions [7] but were met with criticism from some realist evaluators [8, 9]. In this paper, we seek to reflect on (1) the methods and findings of our analyses, identifying where these aligned with realist aims and approaches; (2) the challenges raised about realist trials, whether we encountered these and how we addressed them; and (3) whether or not we were able to conduct a realist trial which generated useful information on what works, for whom, and under what conditions.

## The methods and findings of the INCLUSIVE trial

We have reported analyses of the outcome and process evaluation data using thematic content analysis [10], a variant of grounded theory called dimensional analysis [11], moderation [2], mediation [12], moderated-mediation [13] and qualitative comparative analyses (QCA) [14]. Some publications have explicitly referred to realist evaluation [11, 13, 14] while others have not. Likewise, some of these methods, such as moderator and mediator analyses [15, 16], are controversial within realist circles, while others are not [17–19].

The trial’s overall analysis of primary outcomes reported a significant difference in bullying victimisation at 36-month follow-up between schools allocated to the intervention arm compared to the control, but no difference in aggressive behaviours. In terms of secondary outcomes, the intervention was associated with benefits in terms of quality of life, mental well-being, psychological difficulties, smoking tobacco, drinking alcohol and drug use [2]. These findings are important in terms of overall public health impacts but are not aligned with realist questions on what works for whom, under what conditions and how [15]. Pre-specified subgroup (moderator) analyses showed that LT was more effective for boys, for students who had previously been bullied and for those with higher reported levels of aggression at baseline. However, contrary to our theory of change, no differences in impacts by socio-economic status were found [2]. Such subgroup analyses are common in trials and describe “for whom” health was improved, but are not realist in orientation because they are generally not focused on testing CMOCs. However, in the case of INCLUSIVE, our hypotheses about the intervention having greater impacts among those of lower socio-economic status were based on a CMOC concerning how intervention impacts would be greater among disadvantaged students who are more likely to benefit from an intervention aiming to build commitment to school.

Our next attempt to understand mechanisms involved using a causal-steps mediation analysis [20] to assess whether our a priori theories about whether changes to school organisation, student commitment to school and involvement in anti-school peer groups were implicated in overall mechanisms (but not whether these varied between contexts). Based on our mid-range theory and theory of change, we hypothesised that intervention effects on bullying victimisation at final follow-up (36 months) would be mediated by school climate (as reported by students using the Beyond Blue School Climate Questionnaire [21]), school organisation (as reported by staff using a novel measure created by the INCLUSIVE trial team [22]) and involvement with delinquent peers (as measured by the Young People’s Development Programme measure [23]) all measured at interim follow-up at 24 months. We found that only contact with delinquent peers was impacted by the intervention at 24 months. Intervention impacts on school climate did emerge but not until 36 months. Adjustment for these mediators did not reduce the association between intervention allocation and bullying victimisation, suggesting that, when examining all schools together, there was no evidence of changes in school organisation, student commitment or student involvement in anti-school peer groups being implicated in mechanisms generating outcomes [12]. However, these analyses did not tell us whether these mechanisms might be operating in some schools but not others.

Before we could explore this question of mechanisms operating differently in different schools, we decided we needed to better understand these mechanisms so that we could refine our CMOCs prior to further statistical analyses. Therefore, we undertook analyses of qualitative data to better understand how implementation and mechanisms might vary across schools. We first assessed how implementation fidelity of action groups varied between schools, also exploring how participants described their experiences on action groups, what factors influenced implementation and what consequences they had for schools. We found that in schools where the action group was led by senior staff who were not overwhelmed with other pressures, action groups proved a powerful motor of whole-school change. Having staff-members consistently attend meetings and communicate respectfully with students was described as improving relationships between staff and students, increasing student self-confidence and motivating students to work harder in class [10]. This informed our refinement of CMOCs by helping us understand the ways in which LT resources were used differently in different schools.

We undertook further qualitative analyses to explore mechanisms in more depth and refine our ideas about how and under what conditions these might operate in schools. This involved analysis of qualitative data from case-study schools using “dimensional analysis”, a variant of grounded theory which aims to understand phenomenon in terms of their context, conditions, processes and consequences [24, 25] (a framework with obvious resonance with realist CMOC terminology despite the use of slightly different terms). This analysis identified three mechanisms whereby the intervention might reduce bullying differently in different schools, each consisting of smaller sub-mechanisms. The first mechanisms involved a process of increasing commitment to school by giving students new roles, a forum to share their experiences of being at the school and working with teachers to address shared problems. Such processes could generate consequences of building respectful and warm relationships with staff and increasing students’ sense of belonging at school. This was likely to ensue in conditions in which schools had the capacity and space to engage in such elaborate processes. We thought of these in terms of schools having pre-existing good leadership, less distractions from other problems and a pre-existing inclusive ethos to build on. The second mechanism involved a process of building healthy relationships and behaviours by modelling and teaching pro-social skills via restorative practices, with the consequence of reducing misbehaviour and teaching non-violent conflict management. Such processes required staff who were committed to implementing restorative practice and were more likely to be transformative in schools where most student did not already possess strong pro-social skills. The third mechanism involved a process of de-escalating bullying among a core group of aggressive students via creating a space in which perpetrators could learn about the impacts of their behaviour. Such processes had the consequences of these students learning to empathise, experiencing shame, expressing contrition and accepting responsibility for their actions. Again, such processes were more likely in aggressive or violent schools where committed staff recognised the need and had the capacity to implement restorative practice [11]. Thus, the qualitative data suggested much more detailed ideas about mechanisms and in which schools these mechanisms would generate outcomes [11].

The hypotheses generated through the above qualitative analyses were assessed in two further quantitative analyses using different methods. In the first instance, we used moderated-mediation analyses to explore the first mechanism described above. Specifically, we assessed whether student sense of belonging at interim follow-up might be a mediator of intervention effects on bullying and mental health at final follow-up in schools with certain contextual features. We hypothesised that this would be the case in schools with strong leadership (as indicated by government inspection judgements collected at baseline), low baseline rates of bullying and high baseline student inclusion as measured in our questionnaires. Analysis showed that in schools with these features but not in others, student belonging at interim follow-up did indeed mediate reductions in bullying [13]. Reductions in bullying occurred in other schools but were not mediated by student belonging. Thus, this analysis supported our CMOC that increased student belonging is implicated in mechanisms reducing bullying but only when schools possess the prior capacity, culture and space to promote student belonging via elaborate processes of student engagement. We concluded that, in other schools, other mechanisms, perhaps aligned with mechanisms 2 and 3 described above, were generating reductions in bullying.

In the second instance, we used QCA to explore the complex pathways between allocation to the intervention arm and changes in bullying. Whereas mediation and moderation analyses rely on probabilistic statistics and can only examine the inter-relationships between a small number of variables, QCA instead examines how more complex combinations of multiple conditions appear to enable or preclude the emergence of an outcome, using Boolean logic. A benefit of QCA is that it not only shows the possible pathways to an outcome, but it also shows the pathways that do not lead to the outcome. Our QCA suggested that, as we expected, schools did not need to activate all of the mechanisms identified in the qualitative research to decrease bullying, and that under the correct conditions, often the activation of a single mechanism was sufficient to reduce bullying. Because the data were from a trial with a comparison group of schools, we were also able to explore whether similar mechanisms might occur in schools not in receipt of intervention resources, bolstering our belief that the mechanisms we identified were plausible, transferable, causal and in realist terms, emerging from the realm of the real.

In the next section, we revisit the debate about realist trials and reflect on whether we experienced anticipated challenges and how we addressed them.

## Concerns about realist trials, whether we encountered them, and how we responded

Realist evaluation and critical realism is a broad church with internal disagreements, especially in relation to the use of quantitative data and the usefulness of trials. Central to all interpretations of realism, however, are three interconnected beliefs: reality exists and is independent of human knowledge (ontological realism); knowledge is always incomplete and dependant on the context of its discovery (epistemic relativism); and the rational adjudication between competing claims is possible because reality is intransitive and our means of understanding is transitive and can thus be improved when new knowledge is found (judgmental rationality).

RCTs are not inimical to these tenets of realism. The summary of the studies above suggests that RCTs are a study design which can be used to gather various types of data and analyse these using various methods. While the overall analyses of trial effect sizes clearly do not align with realist evaluations’ concerns with what works, for whom and how, our subsequent analyses suggest that RCTs can nonetheless provide data for analyses which do align with realist concerns, while minimising bias and confounding.

However, several concerns have been expressed by realist evaluators about realist trials, which we now consider. These concerns fall under two key themes. Firstly, some realists regard RCTs as irretrievably positivist in philosophy and reliant on successionism to understand causality, and so are incongruent with realist analysis. Secondly, some realists argue that, in practical terms, RCTs are too narrow in scope to enable realist analyses. Below, we summarise these concerns, consider whether they arose within our trial and describe how we responded to these challenges.

### Concerns about positivism and successionism

The first concern is that RCTs are positivist [9, 15, 26–29]. We have already published a paper on the key tenets of positivism, considering whether or not trials in general are, of necessity or in practice, positivist [30]. We will not repeat those arguments at length but provide a short summary and then consider the case of our own trial. The philosophical and social science literature delineates four key tenets of positivism thus (1) scientific knowledge is derived from direct, sensory observation; (2) theoretical terms must directly equate with empirical measurements with no reference to deeper, unobservable mechanisms of causation; (3) the objective of positivist inquiry is to generate universally applicable laws; and (4) the same methods can be used in the natural and social sciences.

In our previous paper, we argued that, in regard to the first tenet, trials more often use a hypothetico-deductive than an inductive approach, using data not to build theory but rather to assess the falsifiability of hypotheses generated from a priori theory. In response to the second tenet, many trials are theorised purely in terms of the hypothesised association between variables, but this is not a necessary feature. Trials may evaluate interventions informed by theories of change derived from mid-range theory describing deeper mechanisms which need not align with empirical measures. In regard to the third assertion, few trialists claim their results are universally generalisable and many identify factors likely to define the limits of transferability. Some trials, notably realist trials, aim to provide guidance on transferability not in terms of statistical generalisability but via developing or refining theory of how interventions generate certain outcomes in certain settings. Finally, although trials may be used in both the natural and social sciences, trials of social interventions are distinct in their inclusion of qualitative data to explore hermeneutic questions of meaning and agency which are not relevant, for example, in trials of purely natural science (e.g. agricultural) interventions.

Applying these arguments to our own trial, it is clear that INCLUSIVE was hypothetico-deductive in orientation, deriving our hypotheses from a priori theory of change based on a mid-range sociological theory which engaged with the deep mechanisms by which outcomes are generated and were not reducible to associations between empirical constructs. We also aimed to develop findings which might be contingently, but certainly not universally, transferable to other contexts dependent on specific theorised factors. Finally, we used a variety of methods including those rooted in a hermeneutic approach such as interviews, focus groups and semi-structured observations to understand social phenomena.

Related to concerns about trials being positivist (and in particular to the lack of deep theorisation) are some realists’ concerns about how trialists view the world. Realist evaluators commonly argue that trialists think in terms of “interventions working” and are therefore insensitive to the fact that outcomes are actually the result of changes in peoples’ reasoning and actions in response to the availability of novel resources [9, 15]. We acknowledge that trialists (and other evaluators) often write in terms of “intervention X causing outcome Y” but we also believe that this is generally a linguistic short-cut that avoids the consistent wordiness which would be required to remind readers that it is how people employ intervention resources which might generate outcomes.

The debate about realist trials has also revealed a concern relating to positivism, the use of statistical associations between allocation to intervention/control arms, and measures of outcomes as a basis for assessing causality. Although not strictly part of the tenets of positivism, realists have criticised trialists for understanding causation through a “successionist” focus on constant conjunction, arguing that this approach fails to appreciate that, in “open systems”, simple regularities rarely occur [15]:

… events arise from the workings of mechanisms which derived from the structures of objects, and they take place within geo-historical contexts. This contrasts with approaches which treat the world as if it were no more than patterns of events, to be registered by recording punctiform data regarding ‘variables’ and looking for regularities among them... Given the variety and changeability of the contexts of social life, this absence of regular associations between ‘causes’ and ‘effects’ should be expected [16]., pp. 15–16

We agree that trial analyses of overall population effects do not align with realist concerns but counter that other analyses are possible within trial designs that do provide evidence useful for realist questions. We hope that the examples provided above of our own analyses support this point. We would also point out that use of probabilistic statistical measures assessing the associations between two variables (or whether such associations are moderated or mediated by third variables) does not imply a belief that causation can only be considered in terms of constant conjunctions. Indeed, the very use of statistical analyses of the regularity of such conjunctions recognises that these are not constant. The use of statistical moderation analyses in particular reflects a recognition that any conjunctions are contingent on other factors. The use of QCA is also possible within trials, as we have demonstrated, and this rests on an assumption that causality is best assessed by exploring the contingent inter-relationships between multiple factors [19]. Questions about causal attribution are central to trial analyses not because interventions are thought to be the exclusive, determining source of causation but because trials seek to explore how the mechanisms triggered by the introduction of new resources into contexts interacts with all the other mechanisms operating in that context to generate new outcomes. In this sense, trials measure added-value, not unique causation.

The final related concern is about aggregation. Marchal et al. argue that even if a process evaluation attempts to study mechanisms, “such information is lost in the aggregation process required to give RCTs their power” [9] , pp. 125–126. While generating an effect size at the aggregate level is important in trials, the same data sources can be used to answer questions where it would be nonsensical to focus on net-effects. For example, in the abovementioned QCA, we did not aggregate data above the level of individual schools.

### Concerns about the practical feasibility of realist RCTs

The second area of concern relates to whether or not RCTs can practically provide the necessary data to address realist questions. These concerns were difficult to respond to before we finished our analyses, but we are now able to reflect on the challenges we faced and how we addressed them. These concerns include whether randomisation and control stifle our ability to explore CMOCs; RCTs are insufficiently theorised; and trials are only concerned with attribution.

A key concern expressed by those who feel realist RCTs are ill-conceived is that randomisation and control stifle our ability to test CMOCs [8, 9, 15]. This is a significant concern and requires being broken down into its component parts. Firstly, random allocation is important because it ensures that comparisons between intervention and control sites are fair and minimise bias and confounding [13]. As Bonell et al. have previously argued, “Randomisation is merely a practical tool to reduce confounding. It does not fundamentally change the nature of the way we view or research the social world, or affect how we will use comparative empirical data to test hypotheses about mechanisms” [31], pp. 3. Many realist evaluations employ natural experiments which, like RCTs, involve an internal or external comparison group. The only difference in RCTs is that such comparison groups are constructed in such a way that comparisons are balanced. Secondly, control groups are scientifically useful because estimating the effectiveness of interventions is important, and its presence does not diminish our ability to employ other methods or answer other questions.

Another issue related to control is the concern that, in RCTs, the recruitment of participants (individuals or clusters of individuals, for example in schools or villages) is too tightly controlled so that these are insufficiently diverse to allow for cross-contextual comparisons [9], which are necessary for exploring CMOCs. This concern reflects the obviously insufficient diversity in many trials, particularly in biomedical efficacy studies where certain populations are routinely under-recruited or actively excluded [32]. Such homogeneity is not, however, a necessary or desirable feature of RCTs, especially pragmatic trials of public health and other social interventions, where the aim is often to ensure participants reflect the population from which they were recruited. In the INCLUSIVE trial and with the strong support of the funder, we aimed to recruit a diversity of schools and students that reflected the profile of schools and students in England. Our schools were representative across a range of factors including size, population demographic factors, deprivation and educational performance. Participating schools were, however, more likely to have a positive government inspection rating compared to other schools. Within our random allocation of schools to intervention or control group, we stratified randomisation by single-sex versus mixed-sex entry, school-level socio-economic deprivation and student examination attainment to ensure that the trial arms were balanced according to these factors. This stratification meant that, although both intervention and control groups were highly diverse according to these factors, they contained the same range of diversity. This diversity meant that in our moderation and moderated-mediation analyses and QCA, our sample was diverse enough to allow us to explore how indicators of mechanisms and outcomes varied across a diversity of school contexts. This diversity also meant that we could explore contextual contingencies in our qualitative analysis. By purposively selecting schools based on their contextual diversity, we were able to explore what contextual features of a school or community appear to be key to the intervention being implemented (or not), and how and for what purposes intervention resources were used. This allowed us to develop emergent CMOCs [11]. This is not to say that all trials are successful in recruiting diverse samples, but the lack is a weakness in specific studies and not an inherent feature of the RCTs.

Marchal et al. have expressed concerns that RCTs are unable to explore mechanisms and argued, “Even if evaluations of implementation, process, and context are added [to a trial], they can elucidate just that—the intensity, fidelity, and actual process of implementation, and the context in which the intervention took place” [9]. LT was a complex intervention, comprising multiple components and enabling local staff to implement actions appropriate to their school. Our evaluation was therefore built around the assumption that a vast array of mechanisms would be activated by the availability of novel resources because agents would use them in various ways based on their context, which would generate different outcomes in different schools. Our conceptually rich, a priori theory of change facilitated the exploration of these through quantitative and qualitative research. This theoretical underpinning enabled us to identify suitable quantitative measures to include in student and staff surveys. It also enabled us to include suitable prompts to explore in the qualitative data collection guides. This allowed us to focus not only how schools implemented the intervention but also how intervention activities triggered mechanisms in their school. For example, two unanticipated mechanisms to reducing bullying emerged from interviews with students who had participated in restorative conferences: learning empathy and accepting their punishment as fair. These mechanisms were much more likely to activate for students with weaker social skills who benefitted from a more direct lesson in social skills and were less effective in changing behaviour in schools where students knew that their behaviour was unacceptable when they chose to engage in it [11]. Thus, process evaluation data was not just used to study implementation and fidelity, but enabled the discovery and refinement of theorised mechanisms.

## Are realist trials possible and do they generate useful findings?

We believe that the INCLUSIVE trial’s research programme demonstrates that RCTs can provide evidence that is philosophically appropriate for answering realist questions about interventions. Realist trials are possible. The trial we conducted evaluated an intervention with a theory of change based on deep sociological theory of the mechanisms that generate bullying. The trial generated data which allowed for the refinement and testing of CMOCs relating to our intervention. Through it, we discovered that in some schools benefiting from strong management, inclusive cultures and minimal distraction from acute problems, schools could enact complex processes of student involvement which triggered mechanisms of building student belonging in school, in turn generating reductions in bullying and improvements in mental health. In other, more challenged schools, staff implementing processes of restorative practice was sufficient to enable students to develop the skills and attitudes needed to avoid or terminate conflict, which also generated reductions in bullying and improvements in mental health. This allowed us to refine our starting theory of change to provide a much more nuanced picture of what worked for whom and how. Our research also generated nuanced findings which could inform practical intervention modifications, and identify potentially appropriate or inappropriate contexts for intervention transfer.

It is important to note that while some of the aforementioned analyses were explicitly realist, neither the analytic methods we used nor the sorts of questions we sought to explore are unique to realists. For example, researchers on the Aban Aya Youth Project have used growth mixture modelling techniques and found that young men at higher risk of violent trajectories gained the most preventative benefit from a whole-school intervention [33]. Analysis of the KiVa anti-bullying intervention identified both individual and classroom-level mediators which reduced the risk of bullying. By finding that bullies are more likely to offend in contexts where peers encourage violent behaviours [34], researchers were able to specify more clearly how changing peer norms can contribute to decreasing bullying, and incorporated this into new theories and novel interventions. Hence, RCTs need not be explicitly realist in orientation to generate analyses of interest to realists. Nonetheless, we think employing an explicitly realist position does facilitate a more comprehensive assessment of how outcomes are generated by contextually contingent mechanisms.

To incorporate the benefits of realism within the structure provided by a trial, a number of considerations need to be addressed while the trial is being planned. The resources provided in an intervention and the evaluation should both be informed by an intervention theory of change informed by an appropriately selected mid-range theory. Between what Robert Merton called “piecemeal empiricism” and grand theory, mid-range theories are specific to the phenomena of interest but still have sufficient analytic purchase to be generalisable [35]. Based on this mid-range theory, realist trialists need to be explicit about why all intervention resources are being included, and what mechanisms their use is anticipated to activate, and how this varies by population and place. Thus, unlike most conventional theories of change, those used in realist studies must engage with how context and mechanisms interact to generate outcomes. Realist trials should include moderation analyses to shed light on such interactions. Traditionally in trials, moderators examined might include sex, age and socio-economic status, but in realist trials more specific indicators should be included, informed by the theory of change. In the INCLSUIVE trial, these included baseline experiences with bullying and aggression at the individual-level, and school leaderships, ethos, and value-added score at the school-level. As discussed earlier, it is important for realist trials to recruit sufficiently diverse samples of people and/or clusters to explore how context and mechanisms interact to generate outcomes.

Finally, the process evaluations of realist RCTs will focus not only on questions of intervention feasibility, fidelity and acceptability, but also on mechanisms. In a realist trial, diverse stakeholders and participants are asked to describe their context, their positionality, their experiences using intervention resources and what they perceive as having occurred as a result of this use. Realist interviewing, in which participants help develop or refine the study’s logic model [36] can also be used. Participants can be asked about the processes through which they believe change is happening and what the consequences of these processes may be. For example, in INCLSUIVE, instead of speaking about decreasing bullying, students would speak about liking teachers more or getting along better with others in class, which we then theorised would lead to decreased bullying.

There are numerous benefits of incorporating realist approaches into trials. By focusing on what works, for whom, under what conditions and how, trialists’ attention is continually focused on these more specific evaluation questions. However, the benefits also extend beyond evaluation to enable a deeper exploration into the phenomena of interest. While the primary function of INCLUSIVE was to evaluate LT, we also deepened our understanding of bullying, the impact of the school environment on improving mental health and how empathy and forgiveness affect the development of supportive peer-networks. We would argue that our study has demonstrated the value of using RCT designs within realist evaluation. Crucially, randomisation minimises bias and confounding in estimates of intervention effects and moderators, ensuring that we can provide the best possible assessment of what works for whom and under what circumstances.

While detailed findings about the role of context, the activation of mechanisms and explorations of who benefitted from LT were written about in the course of the trial, that does not mean that the study could not have been improved. Despite having context-specific hypotheses in our theory of change, the intervention’s starting logic model was overly simplistic and did not depict differences in how LT was theorised to activate different mechanisms in different contexts. Our logic model did not reflect that one of the objectives of LT as a whole-school intervention was to change the context of the intervention’s implementation throughout the trial. This was especially important because of the variety of schools in the trial. The context at the beginning of the trial for some schools was similar to the context of other schools at final follow-up. Moreover, the logic model was linear, despite acknowledging that complex interventions often contain feedback loops and work in non-linear ways.

Qualitative topic guides would have been improved by focusing more on mechanisms and less on implementation and fidelity, on which we were able to gather from other sources. The baseline and follow-up surveys did not contain measures on all hypothesised mechanisms, such as student-centred framing in relation to teaching and learning. This presented particular challenges with the moderated-mediation and qualitative comparative analyses, in which we either could not explore all the CMOCs which we generated from prior theory and qualitative analyses. However, these limitations were not caused by our use of an RCT design. Despite the challenges arising from insufficient measures or unpredicted mechanisms, the articulation of CMOCs contributed to the development of a broader, realist theory about school environments, bullying as a social phenomenon and how and for whom the introduction of interventions can improve health. Even though we were unable to test all of our hypotheses, they can be used to improve theory and future intervention development.

It is also important to be clear that some of our hypotheses were wrong. Based on the mid-range theory that informed our theory of change, we anticipated greater benefits for socio-economically disadvantaged children [3] but the evidence did not support this [2]. The hypotheses derived from qualitative data and tested using QCA also showed that while participants were able to express a narrative about how LT was used in their school and led to changes, the quantitative evidence did not always bear this out. Indeed, some of the mechanisms that people predicted would be most important had average or no impact [14]. This was not an unexpected finding as within a realist paradigm, knowledge is always partial and perspectival [37].

We deviated from the three-step structure that was proposed in the team’s articulation of what a realist trial would look like [38]. In the original proposal, we planned to create a logic model and theory of change, and use those to develop a priori CMO hypotheses. In phase two, the trial’s process evaluation would be conducted, and the qualitative data would be used to refine those hypotheses. In stage three, data from the process and outcome evaluations would be brought together and the refined CMOs would be tested with moderator and mediator analyses to refine the theory of change. We completed phase one, but our next step was unpacking implementation and assessing contextual variation, and why it was more or less acceptable (and used) by some people and in some places [10]. Rather than simply refine CMOCs in light of qualitative data, we remained sensitised to our theory of change and used dimensional analysis to explore participants’ accounts to build emergent CMOCs [11]. Those CMOCs were then tested using QCA [14]. Separate to this sequential set of analyses, we also ran moderator [2], mediator [12], and moderated-mediational [13] analyses which more closely followed the description of what realist trials might look like. This reflected a complex multi-collaboration acting to generate useful findings at speed. Many of the aforementioned analyses were not part of the trial’s original protocol and were exploratory in nature. Therefore, it would be helpful if future trials of whole-school anti-bullying interventions included analogous analyses into their protocol to assess whether our findings are confirmed.

Finally, it is also important to note that our team’s understanding of realism matured as we carried out this work. In original papers, we wrote that “Realist evaluators have viewed interventions as ‘working’ by introducing *mechanisms* that interact with features of the *context* to produce *outcomes*” [38], pg 2. This was incorrect: resources not mechanisms are introduced into a context.

Despite the aforementioned limitations, we were able to answer detailed questions about how, for whom, under what conditions and to what extent bullying was reduced following the distribution of LT resources to schools in the intervention arm, and what environmental or inter-personal features seemed to affect the generation of those and other outcomes. A simple but important reflection on this process is that RCTs are what researchers make of them. They can be designed to merely assess overall intervention effects, or they can be designed to answer questions which are central to realist enquiry. Most RCTs fall somewhere between those two extremes but crucially, it is not the study design but the detailed planning of theorisation, data collection and analyses that determines what questions a trial may answer.

## Data Availability

Not available
